# A Treatise on the Principles and Practice of Medicine; Designed for the Use of Practitioners and Students of Medicine

**Published:** 1866-04

**Authors:** 


A TREATISE ON THE PRINCIPLES AND PRACTICE OF MEDICINE; DESIGNED FOR THE USE OF PRACTITIONERS AND STUDENTS OF MEDICINE, By AUSTIN FLINT, M.D., Prof. Principles and Practice of Medicine, in the Bellevue Hospital Medical College; and in the Long Island College Hospital; Fellow of the New York Academy of Medicine, etc. Philadelphia: HENRY C. LEA. 1866.

This is a new work on the practice of medicine, by one whose writings are already well known to the profession. The present work consists in one full-sized octavo volume of 876 pages, substantially bound, but printed on too small type for agreeable reading. The first 109 pages are occupied with a discussion of the principles of medicine or general pathology, and the remainder to the special pathology, causes, symptoms, diagnosis, and treatment of individual diseases. It presents a brief, but concise and reliable summary of those pathological and therapeutical views that are most generally accepted by the profession at the present time; and, consequently, it is well adapted for a text-book in the hands of students. It will also form a valuable addition to the library of the practitioner. There are some views presented by the author, in regard to important items of practice, from which we should feel compelled to dissent; some of which we will endeavor to point out hereafter. In the meantime, we cordially commend the book to the attention of our readers.

For sale by W. B. Keen & Co., 148 Lake St.

